# Incidence and severity of nausea and vomiting in a group of maintenance hemodialysis patients

**DOI:** 10.15171/jrip.2017.09

**Published:** 2016-09-03

**Authors:** Mohammad Reza Asgari, Fatemeh Asghari, Ali Asghar Ghods, Raheb Ghorbani, Nahid Hoshmand Motlagh, Fatemeh Rahaei

**Affiliations:** ^1^Nursing Care Research Center, Department of Medical-Surgical Nursing, Faculty of Nursing and Allied Health, Semnan University of Medical Sciences, Semnan, Iran.; ^2^Student Research Committee, Faculty of Nursing, Semnan University of Medical Sciences, Semnan, Iran; ^3^Social Determinants of Health Research Center, Department of Social Medicine, Faculty of Medicine, Semnan University of Medical Sciences, Semnan, Iran; ^4^Department of Allied Health, Faculty of Nursing and Allied Health, Semnan University of Medical Sciences, Semnan, Iran

**Keywords:** Chronic kidney disease, Hemodialysis, Nausea, Vomiting

## Abstract

**Introduction:** Chronic kidney disease (CKD) is a rising problem across the world, including Iran. Most of the patients will require hemodialysis for survival. Despite the great progress has been made in the hemodialysis equipment, but it is still associated with complications. Nausea and vomiting are common complication of during hemodialysis, which leads to unpleasant feeling in patients.

**Objectives:** This study aimed to determine incidence and severity of nausea and vomiting in a group of maintenance hemodialysis patients.

**Patients and Methods:** This is a descriptive and analytical study on 60 hemodialysis patients of dialysis wards in Semnan University of Medical Sciences. Verbal Numeric Rating Scale (VNRS) and Korttila vomiting severity scale were used to measure the severity of nausea and vomiting during hemodialysis respectively.

**Results:** In this study, the incidence of nausea and vomiting during hemodialysis were 28.3% and 11.7%, respectively. Meanwhile, the mean severity of nausea was 1.15 and the mean rank of vomiting was 2.08.

**Conclusion:** The results of the study showed a relatively high incidence of nausea and vomiting in patients undergoing hemodialysis, hence nurses must consider these problems by suitable measures to prevent the occurrence of the these unpleasant feelings in the patients during hemodialysis.

**Implication for health policy/practice/research/medical education:**

Despite the great progress has been made in the hemodialysis equipment, but it is still associated with complications. Nausea and vomiting are common complication of during hemodialysis, which leads to unpleasant feeling in patients. Given the importance of nausea and vomiting developed during hemodialysis and the need to know their incidence in order to evaluate the quality of hemodialysis and plan healthcare policies in this area, the present study was conducted to determine the incidence and severity of nausea and vomiting during hemodialysis in a group patients to provide a more accurate image of hemodialysis quality. The results of the study showed a relatively high incidence of nausea and vomiting in our patients undergoing hemodialysis.

## Introduction

In the past decade, the landscape of health has changed in terms of disease and death due to the advances in science and technology and the changes in lifestyle (1). Communicable and infectious diseases have gotten under control and chronic and metabolic diseases have replaced them (2). Chronic renal failure (CRF) is one of the major causes of death and disability caused by chronic disease across the world (3).

CRF is also referred to as chronic kidney disease (CKD). CKD refers to an advanced and irreversible kidney failure that is often progressive. In general, CKD refers to a condition in which kidney damage or decreased glomerular filtration rate (GFR) persist for three months or longer. This disease eventually leads to end-stage renal disease (ESRD). ESRD is actually the final stage of CKD in which kidney function is no longer adequate to sustain life (4).

The incidence of CKD is increasing across the world for various reasons. On average, 10% of the population suffers from some degree of CKD. In 2000, the global number of CKD patients under treatment across the world was approximately 1.1 million, reaching 2654000 by the end of 2009, revealing an increase of 6%-7%, which is more significant than the global population growth rate. The proportion of hemodialysis patients is estimated to reach 3500000 by 2020 (4).

The growth rate of this disease is about 12% per year in Iran, which is higher than the global average (5). As reported by the Nephrology and Kidney Transplantation Research Center of Iran, about 29000 patients with CKD were under treatment at the end of 2007, 14000 (48.5%) of whom were under hemodialysis (6). At the end of 2011, the number of patients with CKD was about 40000 in Iran (7).

Given that in the final stage of CKD, the kidney function is no longer adequate to sustain life, the patient develops a permanent need for renal replacement therapies at this stage, including dialysis or kidney transplantation (8). In patients with CKD, dialysis is performed through two methods, including hemodialysis and peritoneal dialysis (4). Hemodialysis is the most common treatment used in ESRD. Hemodialysis patients require this particular treatment for the rest of their life or until they receive a successful kidney transplant. Hemodialysis is usually performed 3 times per week and for 3-4 hours every session (9).

Since the second half of the 20th century, hemodialysis has saved the lives of numerous CKD patients and improved their quality of life (10). Although it can lead to increased patient survival, hemodialysis is incapable of altering the natural course of the underlying kidney disease or completely replaces the kidney function. Despite the great advances in hemodialysis technologies, this method is still not without the complications during hemodialysis (11), as CKD and its conservative treatment (i.e. hemodialysis) can lead to many physical, psychological and social problems for the patients (7).

Various complications, including hypotension, muscle cramps, disequilibrium syndrome, nausea and vomiting and dyspnea, with varying degrees of incidence may occur to the patient during hemodialysis, and nurses need to be familiar with the details of these complications. Preventing complications before they develop is of utmost importance. The early detection and modification of life-threatening complications can save the life of patients. Some hemodialysis complications do not threaten the life of the patients but deteriorate their quality of life. Resolving these problems leads to a greater longevity and a better quality of life (4).

Nausea and vomiting are one of the most common complications during hemodialysis. In the study of Chong and Tan on the incidence of gastrointestinal symptoms in Asian patients under regular hemodialysis, anorexia, nausea, vomiting and an early feeling of fullness when eating were experienced to a significant degree. In the study, incidence of nausea and vomiting was reported as 18.2% and 9.8%, respectively (12). Nevertheless, in a study conducted in Iran, the incidence of nausea and vomiting were more common during hemodialysis and up to 25.8% of the cases reported these symptoms (13).

Nausea and vomiting occur for various reasons upon the initiation of hemodialysis. Many patients experience these symptoms during hemodialysis due to the rapid drop in blood pressure or urea (14). Other causes of nausea and vomiting include fever response to municipal water and other materials, disequilibrium syndrome, anxiety, and general causes of nausea and vomiting (4). The multiple etiology of the effects of ESRD and its treatment on the digestive system, the patient’s diet, medication regimen, and developed disabilities are also considered major causes of nausea and vomiting. Fluid overload is also associated with mucosal edema in the gastrointestinal system (along with early satiety), nausea, vomiting, and diarrhea (12).

Nausea and vomiting should be further examined and prevented, as they cause complications during hemodialysis patients. Electrolyte and water imbalance (dehydration) can be considered a major complication of vomiting. The electrolyte and water imbalance following vomiting disrupts the goals of hemodialysis –that is, to maintain a proper and safe concentration of serum electrolytes (4). Nausea and vomiting make dialysis unpleasant for patients and may thus lead to the early discontinuation of dialysis and thereby an undesirable adequacy of dialysis despite the high costs of treatment (15).

## Objectives

Given the importance of nausea and vomiting developed during hemodialysis and the need to know their incidence in order to evaluate the quality of hemodialysis and plan healthcare policies in this area, the present study was conducted to determine the incidence and severity of nausea and vomiting during hemodialysis in our patients so as to provide a more accurate image of hemodialysis quality and to facilitate the devise of plans and strategies for the control and prevention of these complications based on their incidence.

## Patients and Methods

### Subjects

The present descriptive analytical study was conducted on patients selected through convenience sampling. For this purpose, all the eligible patients from the hemodialysis units of hospitals affiliated to Semnan University of Medical Sciences (n=60) were examined in terms of the incidence and severity of nausea and vomiting during hemodialysis. The study inclusion criteria consisted of being sober, not taking any nausea and vomiting medicines from six hours before hemodialysis, having been under hemodialysis for at least three months, age over 20, and requiring 3-4–hour hemodialysis sessions a week.

The questionnaire used consisted of two parts. The first part inquired about the patients’ demographic data, including age, gender, occupation, place of residence, level of education, and duration of dialysis. The second part consisted of a tool for measuring the severity of nausea and vomiting. Nausea was measured by the Verbal Numeric Rating Scale (VNRS). This tool rates the subjects on a scale from 0 to 10, with 0 indicating no nausea and 10 suggesting very severe nausea. The severity of nausea was described with this scale according to the following instructions: A score of zero was taken as the lack of nausea, a score of 1-3 as mild nausea, 4-6 as moderate nausea, 7-9 as severe nausea and 10 as very severe nausea.

The severity of vomiting was determined using the Korttila scale. In this scale, non-retching is taken as the lack of vomiting, retching less than three times with or without gastric emptying as mild vomiting, 3-5 times of retching with or without gastric emptying as moderate vomiting, and more than 5 times of retching with or without gastric emptying as severe vomiting (16). The reliability of the tools was determined through a pilot study of the first 20 subjects using the test-retest method, which was calculated as 97% for the VNRS and 94% for the Korttila scale.

The incidence and severity of nausea and vomiting during hemodialysis were determined in one stage in the patients. At the end of each hemodialysis session and after removing the hemodialysis needle and dressing the sites of vascular access, the researcher visited the patient with a ward nurse (as the project colleague) and explained the objectives and methods of the study to the patient. He then used the VNRS and Korttila scale to measure the patient’s severity of nausea and vomiting during hemodialysis by asking questions and then filled out the questionnaire. To maintain consistent conditions for data collection, it was arranged with the ward head nurse to have the selected patients undergo dialysis at the same place, same shift, and with the same dialysis machine at a temperature of 37°C and with bicarbonate solution. Environmental conditions such as light, sound, smell and temperature were kept consistent during the three sessions. The bed was positioned at a 30-degree angle for all the patients during the study.

### Ethical issues

The research followed the tenets of the Declaration of Helsinki; informed consent was obtained; and the research was approved by the ethical committee of Semnan University of Medical Sciences (Ethical code: 92/372055). Ethical considerations were respected during the study. The researcher first introduced himself to the subjects and briefed them on the study objectives and methods and ensured them about the confidentiality of their data and answered their questions; the subjects then submitted their written consent to participate in the study (Research design code 579).

### Statistical analysis

To analyze the data, Mann–Whitney U test and Spearman’s correlation coefficient were used at a significance level of 5% in addition to calculating the indices of central tendency and dispersion and drawing the frequency distribution tables. The software used was SPSS 18.

## Results

Of all the patients examined, 56.7% (n=34) were male; the mean ± standard deviation (SD) of the patients’ age was 59.5±14.8 years, ranging from a minimum of 23 years and a maximum of 85. A total of 90% of the patients were educated up to high school; 91.7% (n=55) lived in urban areas and 38.3% (n=23) were retired. The mean ± SD of their disease duration was 46.3±42.6 months, ranging from a minimum of four months and a maximum of 209, and with a median of 30.5.

### Incidence and severity of nausea

In line with the study objectives and as shown in Table 1 regarding the incidence and severity of nausea, the incidence of nausea during dialysis was 28.3%, which was mild in 11.7% of the cases, moderate in 13.3%, and severe in 3.3%; none of the patients experienced very severe nausea. The mean severity of nausea was 1.15±2.08 in the patients.

**Table 1:** Absolute and relative frequency of subjects in terms of incidence and severity of nausea during hemodialysis

**Severity of nausea**	**Number**	**Percent**
Lack of nausea	43	71.7
Mild	7	11.7
Moderate	8	13.3
Severe	2	3.3
Very severe	0	0
Mean severity of nausea	1.15 ± 2.08	

### Incidence and severity of vomiting

As shown in Table 2 regarding the incidence and severity of vomiting, the incidence of vomiting was 11.7% in the patients, which was mild in 8.4% of the cases and moderate in 3.3%; none of the patients experienced very severe vomiting. The mean rank of vomiting severity was 2.08 in the patients.

**Table 2:** Absolute and relative frequency of subjects in terms of incidence and severity of vomiting during hemodialysis

**Severity of vomiting**	**Number**	**Percent**
Lack of vomiting	53	88.3
Mild	5	8.4
Moderate	2	3.3
Severe	0	0
Mean rank of severity of vomiting	2.08	

As shown in Table 3 regarding the relationship between the severity of nausea and the demographic variables, a significant relationship was observed between gender and the severity of nausea, as the severity of nausea was higher in women than in men (*P*=0.034); however, no significant relationships were observed between the severity of nausea and age, level of education, place of residence, occupation, and the duration of the disease (*P*>0.05).

**Table 3:** Distribution, mean, SD, median, IQR of severity of nausea in hemodialysis patients according to gender, age, educational level, residential area, job, and disease duration

** Characteristics**	**n**	**Severity of nausea **								**Mean**	**SD**	**Median**	**IQR**	**r**	***P***
		**No Nausea**		**Mild**		**Moderate**		**Severe**							
		**No.**	**%**	**No.**	**%**	**No.**	**%**	**No.**	**%**						
Gender														**-**	0.034
Female	26	15	57.7	4	15.4	6	23.1	1	3.8	1.77	2.45	0.00	4.25		
Male	34	28	82.4	3	8.8	2	5.9	1	2.9	0.68	1.63	0.00	0.00		
Age (y)														-0.220	0.091
<50	15	11	73.3	2	13.3	2	13.3	-	-	0.93	1.71	0.00	2.00		
50-59	14	7	50.0	3	21.4	3	21.4	1	7.1	2.21	2.75	1.00	5.00		
60-69	12	8	66.7	1	8.3	3	25.0	-	-	1.25	1.96	0.00	3.50		
≥70	19	17	89.5	1	5.3	-	-	1	5.3	0.47	1.64	0.00	0.00		
Educational level														0.055	0.677
Illiterate	9	7	77.8	2	22.2	-	-	-	-	0.56	1.13	0.00	1.00		
Primary	22	15	68.2	3	13.6	3	13.6	1	4.5	1.27	2.19	0.00	2.00		
Intermediate	11	10	90.9	1	9.1	-	-	-	-	0.27	0.90	0.00	0.00		
Secondary	12	5	41.7	1	8.3	5	41.7	1	8.3	2.75	2.80	3.00	4.75		
Diploma+	6	6	100	-	-	-	-	-	-	0.00	0.00	0.00	0.00		
Residential area														-	0.756
City	55	39	70.9	7	12.7	7	12.7	2	3.6	1.18	2.12	0.00	2.00		
Rural	5	4	80.0	-	-	1	20.0	-	-	0.80	1.79	0.00	2.00		
Job														**-**	0.257
Housekeeper	22	13	59.1	4	18.2	4	18.2	1	4.5	1.68	2.48	0.00	2.75		
Retired	23	19	82.6	-	-	3	13.0	1	4.3	0.87	2.01	0.00	0.00		
Others	15	11	73.3	3	20.0	1	6.7	-	-	0.80	1.42	0.00	2.00		
Disease duration (mon)														-0.095	0.469
≤12	11	6	54.5	3	27.3	2	18.2	-	-	1.36	1.69	0.00	3.00		
13-24	16	11	68.8	2	12.5	2	12.5	1	6.3	1.38	2.42	0.00	2.75		
25-36	7	6	85.7	-	-	1	14.3	-	-	0.71	1.89	0.00	0.00		
37-60	9	7	77.8	1	11.1	1	11.1	-	-	0.78	1.72	0.00	1.00		
>60	17	13	76.5	1	5.9	2	11.8	1	5.9	1.18	2.38	0.00	1.00		

Abbreviations: IQR, interquartile range; SD, standard deviation.

As shown in Table 4 regarding the relationship between the severity of vomiting and the demographic variables, no significant relationships were observed between the severity of vomiting and any of the studied variables (*P*>0.05).

**Table 4:** Distribution, mean, SD, median, IQR of severity of vomiting in hemodialysis patients according to gender, age, educational level, residential area, job, and disease duration

** Characteristics**	**n**	**Severity of vomiting**								**Mean**	**SD**	**Median**	**IQR**	**r**	***P***
		**No Nausea**		**Mild**		**Moderate**		**Severe**							
		**No.**	**%**	**No.**	**%**	**No.**	**%**	**No.**	**%**						
Gender														**-**	0.102
Female	26	21	80.8	3	11.5	2	7.7	-	-	1.27	0.60	1.00	0.00		
Male	34	32	94.1	2	5.9	0	0.0	-	-	1.06	0.24	1.00	0.00		
Age (y)														0.007	0.956
<50	15	14	93.3	1	6.7	-	-	-	-	1.07	0.26	1.00	0.00		
50-59	14	11	78.6	1	7.1	2	14.3	-	-	1.36	0.74	1.00	0.25		
60-69	12	11	91.7	1	8.3	-	-	-	-	1.08	0.29	1.00	0.00		
≥70	19	17	89.5	2	10.5	-	-	-	-	1.11	0.31	1.00	0.00		
Educational level														0.139	0.290
Illiterate	9	9	100	-	-	-	-	-	-	0.00	0.00	0.00	0.00		
Primary	22	19	86.4	3	13.6	-	-	-	-	1.14	0.35	1.00	0.00		
Intermediate	11	11	100	-	-	-	-	-	-	0.00	0.00	0.00	0.00		
Secondary	12	8	66.7	2	16.7	2	16.7	-	-	1.50	0.80	1.00	1.00		
Diploma+	6	6	100	-	-	-	-	-	-	0.00	0.00	0.00	0.00		
Residential area														-	0.659
City	55	48	87.3	5	9.1	2	3.6	-	-	1.16	0.46	1.00	0.00		
Rural	5	5	100	-	-	-	-	-	-	0.00	0.00	0.00	0.00		
Job														**-**	0.441
Housekeeper	22	18	81.8	2	9.1	2	9.1	-	-	1.27	0.63	1.00	0.00		
Retired	23	21	91.3	2	8.7	-	-	-	-	1.09	0.29	1.00	0.00		
Others	15	14	93.3	1	6.7	-	-	-	-	1.07	0.26	1.00	0.00		
Disease duration (mon)														0.023	0.860
≤12	11	10	90.9	1	9.1	-	-	-	-	1.09	0.30	1.00	0.00		
13-24	16	13	81.3	2	12.5	1	6.3	-	-	1.25	0.58	1.00	0.00		
25-36	7	7	100	-	-	-	-	-	-	0.00	0.00	0.00	0.00		
37-60	9	9	100	-	-	-	-	-	-	0.00	0.00	0.00	0.00		
>60	17	14	82.4	2	11.8	1	5.9	-	-	1.24	0.56	1.00	0.00		

Abbreviations: IQR, interquartile range; SD, standard deviation.

## Discussion

A total of 56.7% of the subjects examined in the present study were male and 43.3% were female. In a study conducted by Ghahri Sarabi et al on dialysis complications and their related factors in hemodialysis patients in select hospitals of Hamadan province, from the total of 192 subjects, 59.9% were male and 40.1% were female (17). Several other studies have also reported a larger number of male patients compared to females (18-22). Unlike the present findings, in a study by Shahdadi et al to compare nausea and vomiting during hemodialysis in the supine position and the half-sitting position, of the total of 45 patients, 64.6% were female and the rest were male (23). Given that hypertension is the second most common cause of CKD (4,24), the risk of ESRD as a result of nephropathy due to hypertension is higher in men than in women (25). Hus et al argued that men have a higher hematocrit level and thus a higher GFR than women and proposed that these higher levels contribute to the greater incidence of kidney dysfunction among men than among women (26).

The majority of the subjects in the present study (51.7%) belonged to the age group of 60 and above and only a minority (8.3%) were below the 20 to 39 age range; the mean age of the subjects was 59.5 years. In a study by Mottahedian-Tabrizi et al, the mean age of the examined hemodialysis patients was 55.05 (18). Accordingly in the study by Monfared et al, most of the patients were over 60 and belonged to the age group of 45-64 (20). Additionally in the study by Ghahri Sarabi et al, the mean age of the subjects was 54.8 and their age range was 20 to 80 (17). Older people are at a greater risk of developing certain kidney and urinary tract diseases such as inflammation of the kidneys, diabetes, urinary tract infections, urinary incontinence, renal artery diseases, and hypertension; as a result, the incidence of CKD increases with age and patients with kidney failure tend to be middle-aged (27). The majority of the subjects examined in the present study (38.7%) were retired and only a minority (1.7%) worked for governmental organizations. In the study of Ghahri Sarabi et al, however, most of the subjects were unemployed (31.3%) and housewives (27.7%) (17). The mean duration of hemodialysis was 46.32 months, and in most of the subjects, this duration exceeded 48 months (26.7%). In the study of Mottahedian-Tabrizi et al, 70% of the patients had a history of hemodialysis for 40-60 months (18). Moreover, in the study by Mirzaei and Azimian, the mean duration of hemodialysis was 31.75 months (13). However, in the study of Ghahri Sarabi et al, most of the subjects had a duration of hemodialysis of less than 2 years with a mean of 4.8 years (17).

The results obtained in this study regarding the incidence and severity of nausea during hemodialysis revealed the incidence of nausea as 28.3% with a mean severity of 1.15±2.08. In the study of Mottahedian-Tabrizi et al, the incidence of nausea during hemodialysis was 33.3% in the subjects with routine nursing care (18), which is higher than the rate reported in the present study. in the study of Ghahri Sarabi et al on the incidence of hemodialysis complications, the incidence of nausea was reported as 9.4% (17). Accordingly in the Chong and Tan study on the incidence of gastrointestinal symptoms in Asian patients undergoing regular hemodialysis reported the incidence of nausea as 18.2% (12), which is lower than the rate reported in the present study.

The results obtained in this study regarding the incidence and severity of vomiting during hemodialysis revealed the incidence of vomiting as 11.7% with a mean rank of vomiting severity of 2.08. In the study of Motahedian Tabrizi et al, the incidence of vomiting during hemodialysis was 16.7% in the subjects with routine nursing care (18), which is higher than the rate reported in the present study. In the study of Sarabi et al on the incidence of hemodialysis complications, the incidence of vomiting was reported as 2.1% (17). In Chong and Tan study on the incidence of gastrointestinal symptoms in Asian patients undergoing regular hemodialysis, the incidence of vomiting was reported as 9.8% (12), which is lower than the rate reported in the present study.

There are different mechanisms at play in the incidence of nausea and vomiting during hemodialysis. Many patients experience this complication during dialysis due to the rapid drop in their blood pressure or urea (14). Other causes of nausea and vomiting include fever response to municipal water and other materials, disequilibrium syndrome, anxiety, and general causes of nausea and vomiting (4). The multiple etiology of the effects of ESRD and its treatment on the digestive system, the patient’s diet, medication regimen, and developed disabilities are also considered major causes of nausea and vomiting. Fluid overload is associated with mucosal edema in the gastrointestinal system (along with early satiety), nausea, vomiting, and diarrhea (12). Nausea and vomiting can also be early signs of disequilibrium syndrome. Both type A and type B dialyzer reactions cause these symptoms. Increased dialysate sodium and calcium may aggravate nausea and vomiting in dialysis patients (24). Eating while on dialysis machine or less than one hour before the start of dialysis can cause nausea during hemodialysis (28). Body position during hemodialysis also affects the incidence of nausea; according to the results obtained by Shahdadi et al, the supine position causes more nausea and vomiting compared to the half-sitting position (23).

The present study revealed a significant relationship between gender and the severity of nausea during hemodialysis, as the severity of nausea was higher in women than in men. No relationships were observed between any of the other variables and the incidence and severity of nausea and vomiting. The proven relationship between fear, anxiety, and depression and the incidence and severity of nausea, and the greater incidence of fear, anxiety and depression in women than in man (29,30) can justify the significant relationship observed between gender and the severity of nausea during hemodialysis.

## Conclusion

There is a relatively high incidence of nausea and vomiting in hemodialysis patients. Nurses are therefore recommended to further consider these complications and to seek appropriate ways to reduce their incidence and severity in hemodialysis patients by performing applied research and evaluating appropriate interventions.

## Limitations of the study

Despite all the efforts made by the researchers, this study had some limitations. One of the limitations was that nausea is a subjective phenomenon and there are no objective criteria for measuring it; the researchers therefore had to rely on the patients’ answers to measure this complication.

## Acknowledgements

This paper is part of a postgraduate thesis (Fatemeh Asghari) that was approved by Semnan University of Medical Sciences under the code 579. Therefore, we would like to express our gratitude to the research deputy of Semnan University of Medical Sciences for all their financial support and also the authorities, nursing personnel and patients of the dialysis units of Semnan Kowsar and Garmsar Emam Khomeini Hospitals for their sincere cooperation. Also we would like to thank the Clinical Research Development Unit of Kowsar Educational and Research and Therapeutic Center of Semnan University of Medical Sciences for providing facilities to this work.

## Conflicts of interest

All authors declare that they have not had any conflicts of interest in conducting the present study.

## Authors’ contribution

MRA, FA and NHM collected the data and re­cruited the patients. RG and FR per­formed the statistical analysis. MRA wrote the paper. AAG edited the final draft. All authors signed the manuscript.

## Ethical considerations

Ethical issues (including plagiarism, misconduct, data fabrication, falsification, double publication, and redundancy) have been completely considered by the authors.

## Funding/Support

This study was supported by Semnan University of Medical Sciences (Grant #579).
